# Pain Across the Menstrual Cycle: Considerations of Hydration

**DOI:** 10.3389/fphys.2020.585667

**Published:** 2020-10-08

**Authors:** Beverly Tan, Michael Philipp, Stephen Hill, Ahmad Munir Che Muhamed, Toby Mündel

**Affiliations:** ^1^School of Sport, Exercise and Nutrition, Massey University, Palmerston North, New Zealand; ^2^School of Psychology, Massey University, Palmerston North, New Zealand; ^3^Advanced Medical and Dental Institute, Universiti Sains Malaysia, Pulau Pinang, Malaysia

**Keywords:** **** pain, menstrual cycle, hydration, drinking, women, hormones

## Abstract

Chronic pain – pain that persists for more than 3 months – is a global health problem and is associated with tremendous social and economic cost. Yet, current pain treatments are often ineffective, as pain is complex and influenced by numerous factors. Hypohydration was recently shown to increase ratings of pain in men, but studies in this area are limited (*n* = 3). Moreover, whether hypohydration also affects pain in women has not been examined. In women, changes in the concentrations of reproductive hormones across menstrual phases may affect pain, as well as the regulation of body water. This indicates potential interactions between the menstrual phase and hypohydration on pain, but this hypothesis has yet to be tested. This review examined the literature concerning the effects of the menstrual phase and hypohydration on pain, to explore how these factors may interact to influence pain. Future research investigating the combined effects of hypohydration and menstrual phase on pain is warranted, as the findings could have important implications for the treatment of pain in women, interpretation of previous research and the design of future studies.

## Introduction

### Human Pain: Why Study It?

Chronic pain – defined as pain that persists for more than 3 months (Treede et al., 2015), is a major health issue with a high and increasing prevalence locally and globally. In New Zealand, 21% of adults reported experiencing chronic pain in 2016, an increase of almost 4 from 17% in 2007 (Ministry of Health, 2016). A survey conducted in the United States estimated that 17% of the population (39 million adults) suffer from chronic pain (Nahin, 2015), while a recent meta-analysis reported a 43% (28 million adults) prevalence of chronic pain in the United Kingdom (Fayaz et al., 2016). The prevalence of chronic pain is expected to continue to rise, especially with an aging population. Apart from its debilitating impact on numerous aspects of the lives of the affected individuals and their families (Turk et al., 2011), chronic pain is also associated with tremendous direct and indirect economic costs. A report estimated the total cost of arthritis in New Zealand in 2010 to be $3.2 billion (1.7% of GDP), with almost half of this amount being attributed to productivity losses alone (Access Economics, 2010). The annual cost of pain in the United States in 2008 was reported to be between $560 and $635 billion dollars, which was more than that for heart disease, cancer, and diabetes (Gaskin and Richard, 2012).

### Human Pain: How to Study It and on Whom?

Pain is a highly subjective and individual experience, with an abundance of physical, biological, and psychosocial factors that independently and interactively influence an individual’s experience of pain (Gatchel et al., 2007; Fillingim, 2017a). Moreover, chronic pain often does not involve actual tissue damage (Jacobs, 2013). The abstract nature of pain presents major challenges to the treatment and management of pain. Although a broad range of pain treatment options are available, they are often very expensive and only minimally effective in eliminating pain and improving quality of life (Martin et al., 2008; Turk et al., 2011). In fact, the average success rate of pain treatments was reported to be only “roughly 30% in about half of treated patients” (Turk et al., 2011). Knowledge and understanding of the underlying mechanisms and factors that contribute to pain is fundamental to the development of more effective pain treatment and management strategies.

Research on pain is typically conducted by inducing acute pain using various experimental modalities in healthy, pain-free individuals. Although recruiting patients with chronic pain as participants would enhance ecological validity of these studies, and is often the target population of interventions etc., this is often difficult to do due to the associated logistical and ethical cost. Acute pain is characterized as being temporary, caused by a specific stimulus and acts as a warning signal (Grichnik and Ferrante, 1991; Świeboda et al., 2013). A wide range of pain stimuli have been used in studies on experimental pain. The more commonly used pain modalities are the cold pressor task, muscle ischemia, mechanical pressure, noxious thermal pain (heat and cold) and electrical stimulation. The type of pain evoked by each of these stimuli differ in a number of characteristics, such as the sensations they produce, pain mechanisms they activate, and more importantly, their resemblance to clinical pain (Rainville et al., 1992; Fillingim and Ness, 2000). Of these pain stimuli, the cold pressor task and muscle ischemia are thought to be the most clinically relevant, as the deep and aching pain produced by both of these stimuli may better replicate the pain experienced by patients with chronic pain (Moore et al., 1979; Rainville et al., 1992; Iacovides et al., 2015a). The responses to these pain stimuli can be assessed using various measures: pain threshold (least amount of a stimulus that elicits feelings of pain), pain tolerance (maximum amount of a painful stimulus that an individual can or is willing to experience) and/or subjective ratings of pain intensity and pain unpleasantness (Edens and Gil, 1995; Hastie et al., 2005; IASP, 2017). Throughout this review, *pain sensitivity* will refer to the outcome of one, or a combination, of these measures.

To date, most of the studies on acute experimental pain have been performed exclusively in male participants, with only a handful performed in women. For instance, among non-human animal studies on pain, 79% of studies that were published from 1996 to 2005 were performed solely in male animals, compared to a mere 8% in female animals (Mogil and Chanda, 2005). In the human literature, a similar bias toward males has been observed across many disciplines, including behavioral research (Beery and Zucker, 2011). Yet, there are clear sex differences in both chronic and acute experimental pain (Unruh, 1996; Berkley, 1997; Riley et al., 1998; Fillingim et al., 2009). In women specifically, the female reproductive hormones and their fluctuation across different menstrual phases can influence pain. Furthermore, these hormones can also affect hydration status – a variable that has recently been implicated as a contributing factor to pain in men, although this has not been investigated in women. Given that women make up half of the global population, it is important to focus specific research on pain in this population. Increased understanding of pain in women could have important implications for the treatment and management of pain, which could ultimately help alleviate the detrimental economic and social consequences of pain.

Several reviews on pain and the menstrual phase have been published (Riley et al., 1999; Sherman and LeResche, 2006; Martin, 2009; Hassan et al., 2014; Iacovides et al., 2015a). However, none of these reviews have addressed the topic with a consideration of hydration status. Therefore, the purposes of this review are to: (i) summarize the existing literature on pain in women, specifically as it relates to the menstrual phase and hydration status, and (ii) make recommendations for future research. A search of the published literature was performed through July 2020 using the PubMed database and Google Scholar search engine, whilst second- and third-order reference lists were checked manually for relevant articles.

## Pain in Women

Sex differences in pain have been extensively studied and research in this area has dominated the pain literature for years (Fillingim et al., 2009). Several comprehensive reviews and meta-analyses on the topic have since been published, which readers are referred to (e.g., Riley et al., 1998; Fillingim et al., 2009; Mogil, 2012; Hashmi and Davis, 2014; Fillingim, 2017b). Overall, there appears to be agreement that women are more sensitive to acute experimental pain than men and many specific chronic pain conditions are also more prevalent among women compared to men (Fillingim et al., 2009; Mogil, 2012). Sex differences in pain modulation pathways, such as opioid analgesia and conditioned pain modulation, have also been reported (Fillingim and Ness, 2000; Paller et al., 2009; Niesters et al., 2010; Popescu et al., 2010; Pisanu et al., 2019). As such, these findings underscore the importance of studying pain in women specifically as it can have implications for the treatment of their pain.

### Overview of Menstrual Cycle Physiology

Plasma concentrations of the main ovarian hormones – estrogen, progesterone, luteinizing hormone (LH) and follicle-stimulating hormone (FSH) – change cyclically during the menstrual cycle. The menstrual cycle, which lasts for an average of 28 days, can be broadly divided into the follicular and luteal phases. The follicular phase begins with the first day of menses and lasts until ovulation (∼ day 14). During this phase, estrogen concentrations initially remain low and stable while menses occurs, increase steadily after cessation of menses, then rise sharply and peak during the last few days of the follicular phase. Progesterone concentrations, on the other hand, remain low throughout the follicular phase. Following the estrogen spike, a mid-cycle surge in LH occurs, resulting in ovulation and the start of the luteal phase. The luteal phase is generally characterized by a rise in progesterone concentrations to its highest point and a concomitant rise in estrogen to moderate concentrations. Toward the end of the luteal phase, both progesterone and estrogen concentrations fall rapidly, resulting in the onset of menses and the start of a new cycle.

Besides these hormonal variations, women also tend to experience fluctuations in several physical and emotional symptoms such as bloatedness, fatigue, irritability, and anxiety over the course of the menstrual cycle (Pfleeger et al., 1997; Alonso et al., 2004). Of importance to this review, recent experimental and clinical data have also shown changes in chronic and acute experimental pain across the menstrual cycle (Martin, 2009; Hassan et al., 2014).

### Acute Experimental Pain and the Menstrual Phase

An early meta-analysis by Riley et al. (1999) and a subsequent review by Martin (2009) both concluded that there are modest effects of the menstrual cycle phase on experimental pain sensitivity in healthy women, although the direction of effects reported by both groups of authors are contradictory. Riley et al. (1999) found lower pain sensitivity in the follicular compared to luteal phase, whereas Martin (2009) concluded that pain sensitivity was *higher* in the follicular phase. In contrast, Sherman and LeResche (2006) reported that the available findings are largely equivocal. Similarly, a recent review of 42 studies by Iacovides et al. (2015a) concluded that the current body of research does not paint a definitive picture on whether the menstrual cycle phase affects experimental pain sensitivity. The conflicting findings are largely due to the numerous methodological variations across studies, such as differences in the experimental pain stimuli used and definition of menstrual phases. Moreover, many studies also did not confirm that participants had ovulated or measure plasma concentrations of estrogen and progesterone to verify menstrual phase, which could subsequently affect the interpretation of study results. These various methodological differences and limitations have been comprehensively reviewed by Sherman and LeResche (2006), which readers are referred to. Interestingly, a slightly greater proportion (64%) of the studies reviewed by Iacovides et al. (2015a) found variations in experimental pain sensitivity across the menstrual cycle. However, the authors noted that the more well-controlled studies mostly did not find any effect of the menstrual cycle phase on pain sensitivity, although these studies were not without some of the methodological limitations as pointed out by Sherman and LeResche (2006). To the best of our knowledge, nine recent studies have been published since the time of the last review in 2015 (Bartley et al., 2015; Iacovides et al., 2015b; Cankar et al., 2016; Palit et al., 2016; Alves et al., 2017; Nayak et al., 2017; Jasrotia et al., 2018; Payne et al., 2019; Pogatzki-Zahn et al., 2019); these findings also appear to be inconsistent. There is a roughly equal number of studies that observed variations in pain sensitivity across the menstrual cycle (5/9 studies) and those that did not (4/9 studies).

A small number of studies have investigated whether the menstrual cycle phase affects experimental pain sensitivity in women with chronic pain conditions. Like the research in healthy women, the findings of these studies are also largely inconsistent. Three studies found variations in experimental pain sensitivity across the menstrual cycle (Isselée et al., 2002; Sherman et al., 2005; Teepker et al., 2011), while four studies did not observe any variability (Alonso et al., 2004; Okifuji and Turk, 2006; Vignolo et al., 2008; Balter et al., 2013).

### Experimental Pain Modulation and the Menstrual Phase

It has been suggested that some of the observed effects of the menstrual phase on experimental pain sensitivity could be related to endogenous pain modulation mechanisms, which consist of pain inhibitory and facilitatory pathways. Pain modulation in humans can be studied experimentally using various methods, with the Conditioned Pain Modulation (CPM) paradigm being the most widely used (Yarnitsky et al., 2010; Lewis et al., 2012; Kennedy et al., 2016). The CPM, which assesses the pain inhibition pathway, involves applying an experimental pain stimulus in one part of the body to dampen the pain produced by another pain stimulus at a different body part (Damien et al., 2018).

To the best of our knowledge, there are nine studies that have examined pain modulation across the menstrual cycle in healthy pain-free women. Seven of these studies assessed pain inhibition using the CPM paradigm (Tousignant-Laflamme and Marchand, 2009; Rezaii and Ernberg, 2010; Bartley and Rhudy, 2012; Rezaii et al., 2012; Wilson et al., 2013; Teepker et al., 2014; Palit et al., 2016). The other two studies used an emotional picture-viewing paradigm that assesses both pain inhibition and pain facilitation. In this method, a series of pictures intended to evoke negative and positive emotions were displayed to participants in order to enhance or reduce, respectively, the perceived intensity of a noxious stimulus (Rhudy and Bartley, 2010; Rhudy et al., 2013). A majority of the CPM studies (5/7 studies) did not observe changes in the magnitude of pain inhibition across the menstrual cycle, while both studies on emotional pain modulation also did not report any menstrual cycle phase effects on emotional pain inhibition or facilitation.

In the only study that investigated pain modulation across the menstrual cycle in women with a chronic pain condition (migraine), no effect of the menstrual cycle phase on CPM inhibition was observed (Teepker et al., 2014).

### Chronic Pain Severity and the Menstrual Phase

In contrast to the uncertainty regarding the menstrual cycle effects on experimental pain sensitivity, studies examining the relationship between the menstrual cycle and chronic pain have produced more consistent results. There are currently two reviews on this topic that have been published. The authors of both reviews found robust evidence indicating that there is menstrual cycle-related variability in the severity of pain symptoms in women with various chronic pain conditions (i.e., migraine, temporomandibular pain disorder, fibromyalgia, rheumatoid arthritis, and irritable bowel syndrome). Moreover, the majority of data appear to show a worsening of self-reported pain severity during the early-follicular and/or late-luteal phase, when plasma estrogen concentrations are low (Martin, 2009; Hassan et al., 2014). However, much of the chronic pain research is also confounded by the various methodological problems and disparities across studies that are present in the experimental pain literature.

### Summary of Pain in Women

Despite the relatively large body of research on the menstrual cycle and experimental pain sensitivity, there is currently no agreement among researchers on whether the menstrual cycle does, or does not, affect experimental pain sensitivity, in both healthy women and those with chronic pain conditions. Regarding pain modulation and the menstrual cycle, the limited number of studies in this area mostly did not observe any menstrual cycle effects on CPM inhibition or emotional pain modulation. In contrast, the severity of pain symptoms for many chronic pain conditions has consistently been shown to vary across the menstrual cycle. Although incompletely understood, the potential mechanisms underlying the variation in pain across menstrual phases could be due to effects of the female reproductive hormones on various pain pathways in the central and peripheral nervous systems, responses to stress and inflammation, and neurotransmitters such as serotonin and α-aminobutyric acid (GABA) (Marcus, 1995; Martin, 2009). However, a discussion on the mechanisms is beyond the scope of this review and interested readers are directed to excellent reviews on this topic (Fillingim and Maixner, 1995; Aloisi and Bonifazi, 2006; Amandusson and Blomqvist, 2013).

The overall ambiguity in this area of research is mostly due to the various methodological inconsistencies and limitations across many of the studies. While a handful of studies have sought to address some of these problems, such as measuring plasma reproductive hormone concentrations and confirming ovulation, there is a paucity of such better-controlled studies. Moreover, none of the previous studies assessed the hydration status of participants, which could be a possible confound.

## Hydration, Menstrual Phase, and Pain

Euhydration is a state of normal body water content, whereas hypohydration refers to the *state* of reduced body water content that exceeds the normal daily fluctuations (>2% body mass loss) (Greenleaf, 1992; American College of Sports Medicine et al., 2007). While there is some variability across studies and individuals, the commonly used biochemical thresholds for defining hypohydration are: serum osmolality (S_Osm_) > 290 mOsm kg^–1^, urine specific gravity (USG) > 1.020 and/or urine osmolality (U_Osm_) > 700 mOsm kg^–1^ (Cheuvront and Sawka, 2005; American College of Sports Medicine et al., 2007; Armstrong et al., 2010, 2012b). Dehydration, on the other hand, refers to the *process* of fluid loss that results in hypohydration (Akerman et al., 2016; Nuccio et al., 2017).

Hypohydration occurs when body fluid losses exceed fluid intake. Excessive fluid losses incurred through sweating (e.g., prolonged exercise, strenuous work, environmental heat exposure) is perhaps the most common way individuals become hypohydrated. This is especially prevalent among athletes, where around 75% of them are already hypohydrated upon arrival for training sessions (Volpe et al., 2009; Arnaoutis et al., 2015; Magal et al., 2015). However, inadequate fluid intake during normal daily activities can also lead to hypohydration. Therefore, hypohydration is also a problem among the general public beyond athletes (Manz and Wentz, 2005; Chang et al., 2016).

### Hypohydration and Pain

Hypohydration has been shown to negatively impact cognitive function, mood state, and fatigue in women (Szinnai et al., 2005; Armstrong et al., 2012a; Pross et al., 2013). These factors, in turn, can contribute to pain (Willoughby et al., 2002), therefore indicating a possible relationship between hypohydration and pain. Indeed, recent research indicates that hypohydration can increase pain. Mild hypohydration of as little as a 1% body mass loss, induced by a combination of fasting and exercise, invoked greater activation in pain-related regions of the brain during an experimental pain task in men (Ogino et al., 2014). Experimental pain sensitivity was also higher when participants were hypohydrated, compared to when they were euhydrated. Similar observations were made in a later study, where a group of men dehydrated by restricting fluid intake for 24 h (Bear et al., 2016). Mild hypohydration (1% body mass loss) was found to increase experimental pain sensitivity relative to the euhydration condition. However, both studies were exclusively performed in men and it is not known whether hypohydration can also contribute to pain in women. In the only study that included female participants, Moyen et al. (2015) conducted a field study on 103 male and 16 female cyclists who were taking part in an ultra-endurance race. Cyclists who were hypohydrated before and during the race reported more intense pain in their leg muscles compared to the euhydrated cyclists. The authors also reported examining possible differences in the pain ratings between the male and female cyclists and did not find sex differences, indicating that hypohydration may also increase pain in women. However, the effect of hypohydration on pain in women has not been formally investigated. This is important as the menstrual phase is associated with variation in body fluid regulation, in addition to their potential impacts on pain as discussed previously.

### Hydration and the Menstrual Phase

One of the more prominent impacts of the menstrual phase on hydration is the osmotic control of arginine vasopressin (AVP) and thirst sensation (Spruce et al., 1985; Vokes et al., 1988; Stachenfeld, 2008). AVP, also known as anti-diuretic hormone, is one of the primary hormones involved in body fluid regulation and its main effect in this context is to increase free water retention in the kidneys (Baylis, 1987). Meanwhile, thirst sensation is the key driver of fluid intake (McKinley and Johnson, 2004). Both AVP and thirst are primarily stimulated by an increase in plasma osmolality (a biomarker of hydration status), such as during dehydration (Baylis, 1987; McKinley and Johnson, 2004).

The luteal phase has been associated with a lowering of the osmotic thresholds at which AVP is released and thirst sensation increases (Spruce et al., 1985; Vokes et al., 1988; Stachenfeld et al., 1999b, 2001) – effects that are primarily attributed to estrogen (Calzone et al., 2001; Stachenfeld and Keefe, 2002). In other words, less of an osmotic stimulus is required to activate the AVP and thirst responses (and their respective effects on increasing renal water retention and fluid intake) during the luteal phase (Giersch et al., 2019). There could also be increased sodium (thus water) retention in the luteal phase, by way of the progesterone-related increased plasma aldosterone concentrations during this phase (Souza et al., 1989; Stachenfeld et al., 1999a, 2001). Moreover, other studies have also observed an increase in body water content measured by bioelectrical impedance analysis during the luteal versus follicular phase (Bunt et al., 1989; Mitchell et al., 1993; Tomazo-Ravnik and Jakopiè, 2006; Fruzzetti et al., 2007; Stachoń, 2016). Therefore, these findings indicate that women may be more protected against dehydration in the luteal compared to follicular phase. Yet, a decreased plasma volume – the fluid component of the blood that is most affected by changes in hydration status – during the luteal phase is also commonly reported (Stephenson and Kolka, 1988; Stachenfeld et al., 1999b, 2001). This is thought to be caused by the preferential movement of fluid from the intravascular space into the interstitium (Øian et al., 1987). Nevertheless, these findings demonstrate that there are differences in body fluid regulation between menstrual phases, which could subsequently affect hydration status.

More importantly, research indicates that hypohydration is a common occurrence among women. A study by Malisova et al. (2016) showed that approximately 20% of the women in Europe met the criteria for hypohydration (U_Osm_ > 810 mOsm kg^–1^). In the United States, data from the third National Health and Nutrition Examination Survey (NHANES III) on nearly 8,000 women showed half of the women to be hypohydrated (plasma tonicity >295 mmom L^–1^) (Stookey, 2005). Furthermore, hypohydration appears to be especially prevalent among the older population. Approximately 30% of older women (aged 50 years and above) were found to be markedly hypohydrated (plasma tonicity ≥300 mmom L^–1^), compared to ∼10% in the younger women (Stookey, 2005). Similarly, Hooper et al. (2015) found that 40% of elderly women living in residential homes met the criteria for hypohydration (S_Osm_ > 295 mOsm kg^–1^), while another study also identified 40% of elderly women to be hypohydrated upon admission to hospital (serum sodium >150 mg dL^–1^ and/or ratio of blood urea nitrogen to creatine >25) (Lavizzo-Mourey et al., 1988).

### Implications of Hydration for Pain in Women

The findings of a hyperalgesic effect of hypohydration in men, and of the potential menstrual phase effects on pain and hydration, have several potential implications for the study and treatment of pain in women. Firstly, hydration status could have confounded previous research on the menstrual phase and pain and contributed to the conflicting findings in the literature. Differences in hydration status between menstrual phases could either exaggerate or mask the menstrual phase effects on pain, resulting in erroneous conclusions about the associations between menstrual phase and pain. However, hydration status was not measured in previous studies on this topic. It may, therefore, be necessary for future research to measure and control for hydration status in order to clarify some of the confusion regarding the effects of the menstrual phase on pain. Secondly, since both the menstrual phase and hypohydration can independently affect pain, they could have interactive effects on pain when combined. In this instance, hypohydration could have a more pronounced hyperalgesic effect in one menstrual phase compared to another. However, this hypothesis has not been investigated. Lastly, hypohydration could reduce the efficacy of pain treatments. For example, Parker et al. (2012) assessed the effect of hypohydration (36 h of fluid restriction) on the outcomes of an osteopathic manipulative treatment program in 8 women and 11 men with chronic low back pain. Greater improvements in treatment outcomes were observed when participants attended the treatment sessions in a euhydrated versus hypohydrated state. Therefore, it may be important for clinicians and practitioners to assess the hydration status of pain patients to maximize the efficacy of the treatments.

## Conclusion

There is currently no definitive conclusion regarding the effects of the menstrual phase on pain. Apart from the methodological limitations and differences (menstrual phase verification, type of experimental pain stimulus etc.) across studies that could explain the conflicting findings, hydration status could also have contributed to the equivocal results in the literature. Hypohydration was shown to increase pain in men, but whether this occurs in women has not been examined. Further, the menstrual phase may influence hydration status. This indicates that hydration status could be a confounding factor in the research on pain in women. Further, hydration status could also influence the pain outcomes, either independently or interactively with the menstrual phase.

Future research should focus on investigating the effects of hypohydration on pain in women across different menstrual phases, including the potential mechanisms that could explain any observed effects. The findings from this research have several important implications: it could (i) help us better understand and interpret previous research on pain in women, particularly as it relates to the menstrual phase, (ii) assist in designing more high-quality research in this area, and (iii) aid in developing strategies to improve the treatment and management of pain in women. The importance and necessity of this research is further underscored by the common occurrence of hypohydration among women. Lastly, while this review focused on premenopausal, normally cycling women, it is also important to address these questions in oral contraceptive users and postmenopausal women due to their different hormonal profiles that could affect their pain responses and/or hydration status (Stachenfeld, 2008; Fillingim et al., 2009; Iacovides et al., 2015a).

## Data Availability Statement

The original contributions presented in the study are included in the article, further inquiries can be directed to the corresponding author.

## Author Contributions

All authors listed have made a substantial, direct and intellectual contribution to the work, and approved it for publication.

## Conflict of Interest

The authors declare that the research was conducted in the absence of any commercial or financial relationships that could be construed as a potential conflict of interest.
